# Ten‐year retrospective review (2003‐2013) of 56 inpatient admissions to stabilize elevated phenylalanine levels

**DOI:** 10.1002/jmd2.12019

**Published:** 2019-03-14

**Authors:** Anne Clark, Christine Merrigan, Ellen Crushell, Joanne Hughes, Ina Knerr, Ardeshir A. Monavari, Eileen Treacy, Aoife Coughlan

**Affiliations:** ^1^ National Centre for Inherited Metabolic Disorders Temple Street Children's University Hospital Dublin Ireland; ^2^ Department of Research Temple Street Children's University Hospital Dublin Ireland

**Keywords:** inpatient admissions, metabolic, phenylketonuria, PKU

## Abstract

Phenylketonuria (PKU) is an inherited metabolic disorder affecting phenylalanine metabolism. The Irish incidence is 1:4500. Currently, there are 500 patients under the care of the National Centre for Inherited Metabolic Disorders in Temple Street Children's University Hospital. Current practice is to admit PKU patients with phenylalanine (phe) levels that are consistently out of range despite an intensive multidisciplinary team input on an outpatient basis. The aim of this study was to evaluate changes in phe levels pre, during, and post admissions and to examine if there was a sustained impact post discharge. Fifty‐six patients were admitted between January 2003 and December 2013. Patients were all <18 years of age. Greater than 70% (n = 39) of the reasons for admission were due to multiple issues. Average admission time was 5 days. There was a significant decrease in median phe levels from prior to the admission to during the admission. However, there was a significant increase in median phe levels from during the admission (505 μmol/L) to both the 1‐6 months' and 7‐12 months' time points (618 and 651 μmol/L, respectively). The results highlight that while inpatient admissions can stabilize levels within the acute setting, this is not sustained long term. The ward environment does not accurately replicate home circumstances. This study highlighted that the reasons for admission are most often multifactorial, which is less likely to be resolved during a brief admission period.

## 1 INTRODUCTION

Phenylketonuria (PKU, OMIM#261600) is an inherited metabolic disorder, which affects the metabolism of the essential amino acid phenylalanine (phe). It manifests from a deficiency of the enzyme phenylalanine hydroxylase, necessary for the conversion of phenylalanine to tyrosine. The resulting elevation in phe can cause significant neurological problems.^1^ The worldwide incidence of PKU is approximately 1:10 000. Within the Republic of Ireland, the incidence is 1:4500.^2^ The National Centre for Inherited Metabolic Disorders (NCIMD) in Temple Street Children's University Hospital (TSCUH) currently has approximately 500 patients with PKU. Clinical management of PKU patients within the NCIMD is to ensure acceptable levels of phenylalanine in order to prevent neurological impairment while maintaining normal growth and development. Prior to July 2017, the NCIMD accepted phe levels between 120 and 400 μmol/L for all age groups with the exception of pregnancy. In pregnancy, accepted phe levels were 150‐250 μmol/L.

Since July 2017, the NCIMD implemented the recommendations of the European Guidelines. These ranges are now set as 120‐360 μmol/L in those aged 12 years and below and 120‐600 mμol/L in >12 years of age, again excluding during pregnancy.^3^ Optimal control is achieved by restricting patient's intake of phe from dietary sources. In order to support adequate growth and development, patients are prescribed a phe‐free supplement that contains essential and nonessential amino acids as well as vitamins and minerals. Poor compliance with one or more variables will cause phe levels to rise outside normal parameters.

Similar to other chronic conditions such as cystic fibrosis and diabetes, the successful management of PKU is dependent upon the acceptance, knowledge, and attitudes of patients and their families/carers.^4^ Acceptance of a PKU diagnosis by patients and subsequent adherence to the low natural protein diet is well documented in the literature to be difficult.^4^, ^5^, ^6^, ^7^, ^8^, ^9^ A 2016 publication by Witalis et al^5^ documented that 63% of children aged between 2 and 10 years and 49% of parents struggled to accept a diagnosis of PKU. A later publication by Witalis et al (2017) further investigating the knowledge and attitudes of parents and children reported that 22% of parents and 30% of children had a sense of helplessness with the daily complexities of trying to implement the low natural protein diet.^6^

Previous unpublished research within the NCIMD has explored potential reasons for elevated phe levels as out lined in Table 1.

**Table 1:** Common Reasons for Elevated Phe Levels

	Common reasons for elevated phe levels
Exchanges	**Persistent excess intake of natural protein (“over exchanging”)** Poor parental comprehension/adherence surrounding counting protein exchanges, older children/teenagers exerting resistance to the diet particularly outside of the home and at social events
Synthetic protein	**Not taking some or all of the prescribed quota of synthetic protein resulting in catabolism** Can be due to transition from one product to another, a negative association with synthetic protein, behavioral reaction to an environmental stimulus
Low protein foods	**Insufficient intake of low protein foods resulting in calorie insufficiency and catabolism** Can be due to difficulties with cooking low‐protein alternatives, fatigue of current products, negative comments from family members/friends regarding low‐protein diet

Above data extracted from patient's individual medical chart, dietetic notes, and phenylalanine‐level sheets.

At present, the NCIMD admits PKU patients whose phe levels are persistently above the recommended range. The decision to admit a patient occurs when other avenues to improve control, such as more frequent monitoring, increased frequency of outpatient reviews with members of the multidisciplinary team (MDT) and consideration to social work involvement have been exhausted. The aim of this project was to retrospectively review phe levels pre, during, and post elective admissions to TSCUH for stabilization of phe levels during the period 2003‐2013.

## 2 PATIENTS AND METHODS

### 2.1 Study sample

Ward admission diaries were used to identify patients admitted for stabilization to the metabolic ward in TSCUH between 2003 and 2013. Patients were between 2 and 18 years of age.

### 2.2 Data collection

All data were recorded from the patients' medical charts and dietetic notes. Demographic data (age and gender) and the reason for admission (poor compliance with synthetic protein, overexchanging, or multiple issues) were also noted. Admissions were either singular (one child admitted) or paired (concurrent sibling and/or matched with a child similar in age). The pairings were mainly children who were previously unknown to each other. The length of stay was reported as the number of the days of admission. Patient's individual phe‐level records were used to collect their phe levels for 6 months prior to admission and during admission from the laboratory system. Post discharge, this was recorded at time points of 1‐6 months and 7‐12 months post discharge, respectively. Compliance at these time points was defined as levels <400 μmol/L.

### 2.3 Statistical analysis

Data were analyzed using the Statistical Package for the Social Sciences (SPSS version 17; Chicago, Illinois) software. A *P* value <0.05 was considered statistically significant.

## 3 RESULTS

Thirty‐six patients had 56 elective admissions to TSCUH during the retrospective study period. Thirty‐one admissions were single patient and 19 admissions were paired. A paired admission is where two patients of similar age with PKU are admitted and educated together.

In Table 2, there were a total of 56 admissions for 36 patients. Some patients had more than one admission. The cohort admitted was largely 2‐11.99 years. Eighteen admissions were for children12‐18 years. The median length of stay was 5 days. There was little difference between males and females who were admitted: 26 males vs 29 females. Thirty‐one of the admissions were single and 19 paired.

**Table 2:** Demographic data

Number of patients	36 Patients
Number of admissions	Total = 56 *(1 exclusion—pregnancy related)* 2‐11.99 years Admissions = 37 12‐18 years Admissions = 18
Length of stay	Median = 5 days (Range 2‐17 days)
Sex	26 male; 29 female
Admission type	31 single admissions; 19 paired admissions

### 3.1 Reason for admission

**Figure 1:**
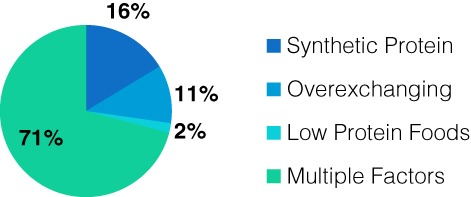
Reason for Admission

The results in Figure 1 show that 71% (n = 37) of the admissions were due to multiple factors. From clinical experience, this incorporates difficulties with synthetic protein, counting exchanges accurately, acceptance of the condition, and parental comprehension. Other admission reasons were identified as singular issues with taking the prescribed amount of synthetic protein (n = 9), over exchanging (n = 6), and adequate intake of low protein foods (n = 1).

### 3.2 Median phe levels at four time points

Table 3 highlights that there was a 14% reduction in median phe levels from prior to admission to 7‐12 months post discharge (*P* = 0.010; 754‐651 μmol/L). Phe levels reduced significantly during admission (*P* < 0.0001; 754‐505 μmol/L). Phe levels increased significantly in the 12 months post discharge (*P* < 0.0001; 505‐651 μmol/L). However, they did not exceed the pre admission levels.

**Table 3:** Median Phe levels at Four Time Points

	Median phe level μmol/L
6 months prior to admission	754
During admission	505
1–6 months post discharge	618
7‐12 months post discharge	651

### 3.3 Percentage of phe levels according to age within range

In Table 4, those patients aged under 12 years maintained better control 7‐12 months post discharge than those who were greater than 12 years old.

**Table 4:** Values using local guidelines

Admission	% Phe levels < 400 μmol/L (under 12's) (n = 37)	% Phe levels < 400 μmol/L (over 12's) (n = 18)
6 months prior to admission	3	0
During admission	22	28
1‐6 months post discharge	14	6
7‐12 months post discharge	16	0

### 3.4 Paired vs single admissions

In Table 5, the mean value of the median phe level prior to admission was 781 μmol/L for the single admissions. It was 764 μmol/L for the paired cohort. At 12 months post discharge, the mean phe value for the single group was 716 (*P* = 0.020). It was 582 μmol/L for the paired cohort (*P* = 0.000).

**Table 5:** Mean value of median phe levels

	Paired admissions μmol/L	Single admissions μmol/L
6 months prior to admission	764	781
During admission	512	497
1–6 months post discharge	584	673
7–12 months post discharge	582	716

## 4 DISCUSSION

Diet therapy is currently the mainstay of treatment for PKU in preventing intellectual disability and to enable patients to achieve their full cognitive and social potential. Despite the success of the diet, compliance and adherence is often dependent upon the understanding, attitudes, and cultural views of the caregivers and patient. During infancy and childhood, this is under the control of parents/guardians. Adolescence and the autonomy that teenagers seek may lead to conflict between parents and children. A study by Jurecki et al^9^ found that patient adherence to recommendations was age‐dependent, decreasing from 88% in children to 33% in adults. Brown and Lichter‐Konecki^10^ found that individuals <18 years were approximately three times more successful in keeping their levels within range than adults.

A study by Witalls et al^5^ investigated the knowledge and attitudes of PKU patients and their caregivers. The study enrolled 173 patients with classical PKU from centers across Poland. The study found that patient's phe intake ranged from 200 to 900 mg (4‐18 exchanges). Synthetic protein ranged from 40 to 80 g/day. The study results indicated that only 45% of children were aware of their daily phe quota. Knowledge surrounding this significantly increased with age. Results from the parental questionnaire highlighted that only 65% were aware of their child's daily phe requirement and 50% were aware of their child's synthetic protein intake. This was despite the majority of the parental cohort residing within socioeconomic affluent areas and having attended third‐level education.

This NCIMD retrospective review is the largest cohort to be studied. A literature search conducted by the authors (Pubmed using key words—Phenylketonuria, Inpatient, and Stabilisation) yielded only one paper, which was a case review by Barr et al.^1^ Barr reported on five patients with classical PKU requiring strict dietary restriction. The reason for admission for the five patients seemed to be multifactorial with the exception of one patient whose noncompliance with synthetic formula was the principal issue. Barr et al found that the focused and controlled hospital setting demonstrated an improvement in phe levels. However, this was not maintained post discharge.

Other centers have looked at methods outside of traditional education that is mainly clinic‐based. Singh et al^11^ used a camp experience and targeted teenage girls. Positive short‐term effects were achieved. These were not sustained in the long term. Freehauf et al^12^ reported on a 2‐day conference targeted at children 10 years and older. The conference was diverse in content from practical cookery sessions to a motivational speaker.

At present, the challenge for the NCIMD is managing the blood phe levels of a large patient cohort using a paper‐based system. Paper‐based phe‐level sheets make it difficult to keep track of trends. It also fails to alert when levels are not being sent and those who do not make contact to discuss levels outside of the recommended reference range. A paperless system with flags/alerts when levels are out of range would assist with managing this patient group and flag early those patients whose metabolic control is declining.

Education in the supportive ward environment by the MDT does not replicate the home situation/environment. These patients are not sick and admission times were on average 5 days (Table 2). This is an insufficient time to assess, provide education, and allow for improvement when reasons for admission were largely multifactorial, as demonstrated in Figure 1. This is further demonstrated in Table 3. Phe levels increased significantly in the 12 months post discharge. However, the levels did not exceed those from preadmission. The challenge of maintaining levels during and post admission is challenging regardless of age, as demonstrated in Table 4.

The ward environment, with other children who are unwell, is not the best setting to improve and optimize metabolic control in PKU. As highlighted by Singh et al^11^ and Barr et al,^1^ the desired metabolic control gradually disappeared following their interventions. Our review showed a similar effect post ward admission, as highlighted in Table 5. However, our review showed that while both groups had a statistically significant reduction, the paired group achieved a more clinically significant reduction (24% reduction vs 8.3% reduction). This then brought the mean phe levels of the paired group closer to the therapeutic range. Control, where achieved, was not fully sustained in the medium‐to‐long term. The Cochrane Review of Dietary Interventions for Phenylketonuria in 2010^13^ concluded that more research is needed to show that it is safe to relax diet later in life. Until this is proven, “diet for life,” which has always been the practice in Ireland, will remain as the mainstay of treatment in PKU. Bosch et al carried out a study looking at the impact of PKU on health‐related quality of life.^14^ A negative impact of PKU from an emotional point of view was found.

Therefore, it is important to look at novel approaches to maintaining long‐term phe control in patients who have required hospital admissions. Examples to consider would be online education sessions, for example, webinars or Skype. The authors of the study acknowledge the need for further research within this area particularly looking at the qualitative effects of admissions on patients and their caregivers. Additionally, if patients continue to be admitted to hospital, there needs to be a structured format to intervention including a policy for the admission of PKU patients requiring MDT management. This approach requires follow through post discharge to improve longer‐term control.

## 5 CONCLUSION

The results highlight that ward admissions are not a long‐term solution to poor metabolic control in PKU. We suggest that other and novel forms of intervention, for example, webinars, Skype sessions, apps, or structured outpatient sessions, need to be investigated.

## CONFLICT OF INTEREST

The author(s) declared no potential conflicts of interest and received no financial support with respect to the audit, authorship, and publication of this article.
